# Big Five Traits as Predictors of a Healthy Lifestyle during the COVID-19 Pandemic: Results of a Russian Cross-Sectional Study

**DOI:** 10.3390/ijerph191710716

**Published:** 2022-08-28

**Authors:** Alena Zolotareva, Sergei Shchebetenko, Svetlana Belousova, Irina Danilova, Vadim Tseilikman, Maxim Lapshin, Lilia Sarapultseva, Svetlana Makhniova, Maria Sarapultseva, Maria Komelkova, Desheng Hu, Shanshan Luo, Ekaterina Lisovskaya, Alexey Sarapultsev

**Affiliations:** 1 School of Psychology, HSE University, 101000 Moscow, Russia; 2 Russian-Chinese Education and Research Center of System Pathology, South Ural State University, 454080 Chelyabinsk, Russia; 3 Department of Psychology, Chelyabinsk State University, 454001 Chelyabinsk, Russia; 4 Institute of Natural Sciences and Mathematics, Ural Federal University Named after the First President of Russia B.N. Yeltsin, 620002 Ekaterinburg, Russia; 5 Institute of Immunology and Physiology, Ural Branch of the Russian Academy of Science, 620049 Ekaterinburg, Russia; 6 School of Medical Biology, South Ural State University, 454080 Chelyabinsk, Russia; 7 Department of Mathematics and Natural Sciences, Russian State Vocational Pedagogical University, 620012 Ekaterinburg, Russia; 8 Department of Pediatric Dentistry, Medical Firm Vital EBB, 620144 Ekaterinburg, Russia; 9 Department of Integrated Traditional Chinese and Western Medicine, Union Hospital, Tongji Medical College, Huazhong University of Science and Technology, Wuhan 430022, China; 10 Institute of Hematology, Union Hospital, Tongji Medical College, Huazhong University of Science and Technology, Wuhan 430022, China

**Keywords:** healthy lifestyles, personality traits, COVID-19 pandemic

## Abstract

The healthy lifestyle of people around the world has changed dramatically during the COVID-19 pandemic. The personality risk factors for these processes from around the world remain understudied. This study aimed to examine the associations of the Big Five traits with a healthy lifestyle during the COVID-19 pandemic. In a cross-sectional study, data from 1215 Russian university students were analyzed. Participants completed the Big Five Inventory-10 and Short Multidimensional Inventory Lifestyle Evaluation. The results showed that personality traits predicted many dimensions of a healthy lifestyle during the COVID-19 pandemic. Diet and nutrition were positively predicted by extraversion, agreeableness, and conscientiousness, and it was negatively predicted by neuroticism. Substance abuse was positively predicted by agreeableness and conscientiousness, and it was negatively predicted by extraversion. Physical activity was positively predicted by extraversion and conscientiousness, and it was negatively predicted by neuroticism. Stress management was positively predicted by extraversion and conscientiousness, and it was negatively predicted by neuroticism. Restorative sleep was positively predicted by extraversion and conscientiousness, and it was negatively predicted by neuroticism. Social support for healthy practices was positively predicted by extraversion, agreeableness, and conscientiousness. Environmental exposures were positively predicted by extraversion, and neuroticism was positively and negatively predicted by conscientiousness. Our findings may be useful for further exploration of personality risk factors for healthy practices in challenging life circumstances.

## 1. Introduction

A healthy lifestyle can be defined as complex behavior patterns and routines that include healthy practices and exclude harmful habits. Healthy practices imply balanced nutrition and high consumption of fruits and vegetables, regular physical exercises and activities [1], rest, and restful sleep [2]. On the contrary, harmful habits involve poor eating [3], physical inactivity and sedentary behavior [4], sleep deficiency and deprivation [5], alcohol consumption [6], excessive smartphone use [7], and active, passive, and electronic smoking [8]. A healthy lifestyle has been associated with increased life expectancy [9], mental health [10], and psychological well-being [11], as well as beneficial effects on the hormonal [12], immune [13], and hematopoietic systems [14].

Many people went through significant transformations in their healthy lifestyles throughout COVID-19. At the beginning of the pandemic, the average number of steps dropped from 10,000 to 4600 steps per day, sleep increased by 25–30 min per night, social time increased by more than half to less than 30 min, and screen time increased by more than double to more than 5 h per day [15]. Previous studies revealed unfavorable immediate and long-term impacts of the pandemic on self-reported physical activities and sedentary behaviors among people [16], including changes in eating habits [17], increased substance use [18], and problematic smartphone use [19]. These led to subsequent weight gain in more than a third of the people surveyed in France [20], Italy [21], Poland [22], UAE [23], and many other countries.

Importantly, individuals with unhealthy lifestyle patterns have been reported to have more severe forms of COVID-19 [24]. The mechanisms that underpin these relations are not fully understood. They may include the activation of the immune and stress systems, as well as the detrimental influence of obesity on the course of the disease [11,25,26]. Unhealthy lifestyles can also lead to reduced immunity and organ injury, predisposing people to diseases, and their severity, commonly defined as ‘communicable’ such as SARS-CoV-2 [27].

Lifestyle changes during lockdown occurred in both unfavorable and favorable directions. According to the results of the French NutriNet-Santé cohort study, 19.8% of the participants reported positive changes, such as increased consumption of fruits, vegetables, and fish [20], while increased physical activity, especially for bodyweight training, was reported by 38.3% of respondents from Italy [21]. In children and adolescents, the adverse effects of SARS-CoV-2 include an unbalanced diet with an increased risk of overweight or nutritional deficiencies, a sedentary lifestyle, lack of schooling, social isolation, and deteriorating mental health [28].

Although a healthy lifestyle was critical during the COVID-19 pandemic, personality risk factors have not yet been investigated. In pre-pandemic studies, personality traits have been associated with health [29,30,31]. Physical activity and a vegetable diet were associated with increased extraversion, adherence to the Mediterranean diet, and lower alcohol and cigarette intake, and excessive use of screens and social networking sites was associated with greater conscientiousness. Lower fruit consumption, sleep issues, alcohol and tobacco consumption, smartphone use, and excessive use of the internet were all associated with increased neuroticism. Likewise, according to Zhu et al. (2021), greater perceived vulnerability, feeling stressed, apprehensive, and helpless during the pandemic was associated with more reports of positive lifestyle changes, including increased social/family support, increased awareness of mental health, and a positive lifestyle [32].

Given the above, this study aimed to examine possible associations of the Big Five traits with a healthy lifestyle during the COVID-19 pandemic.

## 2. Materials and Methods

### 2.1. Procedure and Participants

The data were collected in March and April 2021. We invited students from four Russian universities in Yekaterinburg and Chelyabinsk to participate in this study. We conducted the study in a psychology class, with 1236 students volunteering. Subsequently, 21 questionnaires were excluded due to incomplete completion, and data from different universities were combined because the procedure and methods of diagnosing COVID-19 disease, the patients’ routing protocols in case of positive results, and the algorithms for inpatient and outpatient treatment were the same throughout the country (in accordance with the orders of the Ministry of Health).

The total sample consisted of 1215 university students (71.9% females) aged 18–26 (M = 19.37, SD = 1.32). All volunteers completed a written informed consent form, disclosing the purposes of this study and stating that they could withdraw from participation at any time. The study was approved by an Institutional Review Board at Chelyabinsk State University (# 6, 17 November 2020) and carried out in accordance with the Declaration of Helsinki [33].

### 2.2. Measures

#### 2.2.1. Personality Traits

To measure personality traits, we used a Russian version [34] of the Big Five Inventory-10 (BFI-10) [35]. The BFI-10 consists of 10 items evaluating 5 personality traits: extraversion (e.g., “…is outgoing, sociable”), agreeableness (e.g., “…is generally trusting”), conscientiousness (e.g., “… does a thorough job”), neuroticism (e.g., “…gets nervous easily”), and openness to experience (e.g., “…has an active imagination”). Respondents rated their agreement using a five-point Likert scale. The internal consistency was evaluated using Spearman–Brown coefficients. In the current study, the Spearman–Brown coefficients were 0.67, 0.25, 0.47, 0.64, and 0.30 for extraversion, agreeableness, conscientiousness, neuroticism, and openness to experience, respectively.

#### 2.2.2. A Healthy Lifestyle

To measure a healthy lifestyle, we used a translated Russian version of the Short Multidimensional Inventory Lifestyle Evaluation (SMILE-C) [36]. SMILE-C consists of 27 items that examined 7 dimensions of a healthy lifestyle during the COVID-19 pandemic: diet and nutrition (e.g., “Do you eat healthy foods such as fresh fruits, fresh vegetables, whole grains, legumes, or nuts?”), substance use (e.g., “Do you use marijuana or hashish?”), physical activity (e.g., “Do you exercise for at least 30 min daily (or 150 min a week)?”), stress management (e.g., “Do you make time to relax?”), restorative sleep (e.g., “Do you manage to sleep between 7 and 9 h per night?”), social support (e.g., “Do you interact with your friends and/or relatives?”), and environmental exposures (e.g., “Do you spend time on a computer/smartphone within one hour of going to sleep?”). Respondents rated their agreement using a four-point Likert scale. Higher scores indicated a healthier lifestyle. In the current study, Cronbach’s alpha was 0.47, 0.56, 0.60, 0.53, and 0.80 for diet and nutrition, substance use, stress management, restorative sleep, and social support, respectively. The physical activity and environmental exposure scores were measured using a single question.

### 2.3. Data Analysis

All analyses were performed using software IBM SPSS version 27 (IBM Corporation, Armonk, NY, USA). In the first stage, descriptive statistics and correlation matrix for all study variables were calculated. In the second stage, multivariate regression analyzes were used to examine personality risk factors for a healthy lifestyle during the COVID-19 pandemic.

## 3. Results

### 3.1. Preliminary Analyses

Table 1 summarizes the correlation matrix for all study variables.

Personality traits were correlated with many healthy lifestyles. Therefore, extraversion was negatively associated with substance use and positively associated with diet and nutrition, physical activity, stress management, restorative sleep, and social support for healthy practices. Agreeableness was positively related to diet and nutrition, substance use, and social support for healthy practices. Consciousness was negatively correlated with environmental exposures and positively correlated with diet and nutrition, substance use, physical activity, stress management, restorative sleep, and social support for healthy practices. Because higher SMILE-C scores indicated a healthier lifestyle, individuals with increased conscientiousness and agreeableness showed decreased substance use. Neuroticism was positively related to environmental exposures and negatively related to diet and nutrition, physical activity, stress management, restorative sleep, and social support for healthy practices. Openness to experience was positively correlated with physical activity, stress management, and social support for healthy practices.

### 3.2. Regression Analyses

We tested linear regression models that predict changes in COVID-19 on seven lifestyle indicators separately (Table 2).

Extraversion, agreeableness, and conscientiousness were positively predicted while neuroticism negatively predicted diet and nutrition, R^2^ = 0.11, F (5, 1209) = 32.14, *p* < 0.001. Substance abuse was positively predicted by agreeability and conscientiousness and was negatively predicted by extraversion, R^2^ = 0.05, F (5, 1209) = 11.64, *p* < 0.001. Extraversion and conscientiousness positively predicted while neuroticism negatively predicted physical activity, R^2^ = 0.09, F (5, 1209) = 24.40, *p* < 0.001. Stress management was positively predicted by extraversion and conscientiousness, and negatively predicted by neuroticism, R^2^ = 0.20, F (5, 1209) = 58.61, *p* < 0.001. Extraversion and conscientiousness positively predicted while neuroticism negatively predicted restorative sleep, R^2^ = 0.07, F (5, 1209) = 17.77, *p* < 0.001. Social support for healthy practices was positively predicted by extraversion, agreeableness, and conscientiousness, R^2^ = 0.18, F (5, 1209) = 54.06, *p* < 0.001. Extraversion and neuroticism were positively predicted while conscientiousness negatively predicted environmental exposures, R^2^ = 0.03, F (5, 1209) = 6.82, *p* < 0.001. Therefore, openness to experience was the only variable that did not incrementally predict a healthy lifestyle during the COVID-19 pandemic.

## 4. Discussion

This study reveals that personality traits predicted various indicators of a healthy lifestyle during the COVID-19 pandemic. Extraversion, agreeableness, and conscientiousness were found to be favorable predictors of numerous aspects of a healthy lifestyle, while neuroticism was found to be a negative predictor. There was one exception to these findings. Extraversion was found to be a negative predictor of substance use. In other words, the higher the extraversion score, the less likely people were to use alcohol, tobacco, or psychoactive substances. Similar findings have previously been obtained in the areas of stimulant use, smoking, alcohol consumption, and opioid dependency [37,38,39]. Higher extroversion was also associated with a perceived decrease in alcohol consumption and a shift to a healthier diet among Finnish women during the COVID-19 pandemic [40]. Furthermore, during the COVID-19 pandemic, receptivity to new experiences did not predict a healthy lifestyle. This finding supports previous research that found no significant links between openness to experience and hedonistic lifestyles among university students [41] or health behaviors among young people [42].

Personality traits largely determine the degree to which people adapt to the pandemic, although it appears that personality characteristics can be the basis for healthy lifestyles regardless of COVID-19. Thus, lower neuroticism and higher extraversion, agreeableness, and consciousness were associated with improved mood, decreased perceived stress, and increased participation in health promotion among college students both before and after the onset of the pandemic [43]. A recent large-scale panel study of the British population (UK Household Longitudinal Study, UKHLS) found that during the pandemic, people with more extroverted and open personality traits reported greater deterioration in mental health, while those with higher agreeableness scores suffered less [44]. Neuroticism significantly predicted cross-sectional mental health deterioration but did not significantly worsen during the pandemic. Similar to previous findings, our study demonstrated a sustained role of personality traits in healthy lifestyles and mental health, which is intensified in times of crisis such as COVID-19.

This study has several strengths and limitations. In contrast to previous studies, our findings have expanded ideas about the relationship between personality traits and healthy lifestyles during the COVID-19 pandemic. In addition to alcohol consumption [40], our study covered many other aspects of a healthy lifestyle. As for limitations, the nature of the study’s self-report could distort the objective picture of healthy practices and harmful habits. Previous studies showed that self-reporting underestimated sedentary behavior compared to device measurements [45], underreported energy intake, especially when the body mass index of the respondents’ increases [46], and underestimated smoking behavior compared to biomarkers [47]. Next, this study is cross-sectional, which limits the possibility of causal inferences from the findings obtained, although the regression analysis is more suitable for longitudinal data [48]. Moreover, we employed a convenience sample of Russian undergraduates, which could distort revealed associations to a more global population. Some concerns are also raised by the fact that the study sample was only students from four Russian universities in two nearby cities. In this regard, we understand that our findings may be explained to some extent by geographic characteristics. Finally, we did not control for the experience of COVID-19 disease, but this factor could have influenced our findings.

## 5. Conclusions

This study highlighted the relationship between the five main characteristics and a healthy lifestyle during the COVID-19 pandemic. Our findings extend previous research on the relationship between personality traits and healthy practices by describing this relationship in challenging life circumstances. Although this relationship needs to be tested in longitudinal studies, we anticipate that our findings will help scientists and practitioners better understand and adjust personality risk variables for healthy behaviors. Specifically, we suggest that preventive measures and psychoeducational activities may be useful for young people with personality traits responsible for unhealthy lifestyles.

## Figures and Tables

**Table 1:** Correlation matrix for study variables.

Model	Ext	Agr	Con	Neu	Ope
Diet and nutrition	0.18 ^a^	0.13 ^a^	0.29 ^a^	−0.16 ^a^	0.04
Substance abuse	−0.08 ^b^	0.12 ^a^	0.13 ^a^	0.05	0.02
Physical activity	0.19 ^a^	0.01	0.25 ^a^	−0.12 ^a^	0.07 ^c^
Stress management	0.34 ^a^	0.06	0.32 ^a^	−0.19 ^a^	0.10 ^a^
Restorative sleep	0.14 ^a^	0.05	0.20 ^a^	−0.17 ^a^	0.01
Social support	0.40 ^a^	0.15 ^a^	0.20 ^a^	−0.10 ^a^	0.10 ^a^
Environmental exposures	0.05	0.01	−0.11 ^a^	0.10 ^a^	−0.02

Note: ^a^ *p* < 0.05, ^b^ *p* < 0.01, ^c^ *p* < 0.001. Ext = Extraversion, Agr = Agreebleness, Con = Conscientiousness, Neu = Neuroticism, Ope = Openness to experience.

**Table 2:** Linear regression models that predict a healthy lifestyle during the COVID-19 pandemic.

Model	B	SE	β	t	*p*
Model 1: Risk factors for diet and nutrition					
**Extraversion**	**0.11**	**0.03**	**0.09**	**3.27**	**0.001**
**Agreeableness**	**0.13**	**0.04**	**0.10**	**3.48**	**0.001**
**Conscientiousness**	**0.34**	**0.04**	**0.25**	**8.82**	**<0.001**
**Neuroticism**	**−0.13**	**0.03**	**−0.11**	**−4.09**	**<0.001**
Openness to experience	0.01	0.04	0.01	0.21	0.838
Model 2: Risk factors for substance abuse					
**Extraversion**	**−0.12**	**0.03**	**−0.12**	**−4.05**	**<0.001**
**Agreeableness**	**0.15**	**0.03**	**0.13**	**4.41**	**<0.001**
**Conscientiousness**	**0.17**	**0.03**	**0.15**	**5.05**	**<0.001**
Neuroticism	0.05	0.03	0.05	1.70	0.089
Openness to experience	0.02	0.03	0.01	0.49	0.627
Model 3: Risk factors for physical activity					
**Extraversion**	**0.07**	**0.01**	**0.13**	**4.67**	**<0.001**
Agreeableness	−0.02	0.02	−0.03	−1.19	0.234
**Conscientiousness**	**0.12**	**0.02**	**0.22**	**7.59**	**<0.001**
**Neuroticism**	**−0.04**	**0.01**	**−0.08**	**−2.73**	**0.007**
Openness to experience	0.02	0.02	0.04	1.38	0.168
Model 4: Risk factors for stress management					
**Extraversion**	**0.43**	**0.04**	**0.26**	**9.79**	**<0.001**
Agreeableness	−0.01	0.05	−0.01	−0.12	0.907
**Conscientiousness**	**0.46**	**0.05**	**0.24**	**9.02**	**<0.001**
**Neuroticism**	**−0.20**	**0.04**	**−0.13**	**−4.83**	**<0.001**
Openness to experience	0.08	0.05	0.05	1.82	0.070
Model 5: Risk factors for restorative sleep					
**Extraversion**	**0.09**	**0.03**	**0.08**	**2.66**	**0.008**
Agreeableness	0.03	0.04	0.02	0.72	0.474
**Conscientiousness**	**0.23**	**0.04**	**0.17**	**5.86**	**<0.001**
**Neuroticism**	**−0.15**	**0.03**	**−0.14**	**−4.88**	**<0.001**
Openness to experience	−0.02	0.04	−0.02	−0.53	0.597
Model 6: Risk factors for social support					
**Extraversion**	**0.62**	**0.05**	**0.35**	**12.94**	**<0.001**
**Agreeableness**	**0.20**	**0.05**	**0.10**	**3.67**	**<0.001**
**Conscientiousness**	**0.22**	**0.05**	**0.11**	**4.10**	**<0.001**
Neuroticism	−0.05	0.05	−0.03	−1.00	0.317
Openness to experience	0.08	0.05	0.04	1.51	0.131
Model 7: Risk factors for environmental exposures					
**Extraversion**	**0.03**	**0.01**	**0.09**	**3.08**	**0.002**
Agreeableness	0.01	0.01	0.01	0.16	0.870
**Conscientiousness**	**−0.05**	**0.01**	**−0.11**	**−3.83**	**<0.001**
**Neuroticism**	**0.04**	**0.01**	**0.11**	**3.68**	**<0.001**
Openness to experience	−0.01	0.01	−0.02	−0.85	0.397

Note: Significant predictors are marked in bold.

## Data Availability

The datasets analyzed during the current study are available from the corresponding author upon reasonable request.
